# Correction to: Regulation of Carotenoid Biosynthesis by Shade Relies on Specific Subsets of Antagonistic Transcription Factors and Cofactors

**DOI:** 10.1093/plphys/kiac120

**Published:** 2022-03-25

**Authors:** Jordi Bou-Torrent, Gabriela Toledo-Ortiz, Miriam Ortiz-Alcaide, Nicolas Cifuentes-Esquivel, Karen J Halliday, Jaime F Martinez-García, Manuel Rodriguez-Concepcion

*Plant Physiology*, https://doi.org/10.1104/pp.15.00552

In the originally published version of this manuscript, a funding statement was omitted from the Acknowledgements. The Acknowledgements should have included the following statement: “MRC was funded by project BIO2014-59092-P funded by MINECO/AEI and FEDER.”

These details have been corrected only in this correction notice to preserve the published version of record.

